# Inflammatory Responses Regulating Alveolar Ion Transport during Pulmonary Infections

**DOI:** 10.3389/fimmu.2017.00446

**Published:** 2017-04-18

**Authors:** Christin Peteranderl, Jacob I. Sznajder, Susanne Herold, Emilia Lecuona

**Affiliations:** ^1^Department of Internal Medicine II, University of Giessen and Marburg Lung Center (UGMLC), Member of the German Center for Lung Research (DZL), Giessen, Germany; ^2^Division of Pulmonary and Critical Care Medicine, Feinberg School of Medicine, Northwestern University, Chicago, IL, USA

**Keywords:** ion channel, ion pumps, edema, cytokines, Na-K-ATPase, cystic fibrosis membrane conductance regulator, epithelial sodium channel, lung injury

## Abstract

The respiratory epithelium is lined by a tightly balanced fluid layer that allows normal O_2_ and CO_2_ exchange and maintains surface tension and host defense. To maintain alveolar fluid homeostasis, both the integrity of the alveolar–capillary barrier and the expression of epithelial ion channels and pumps are necessary to establish a vectorial ion gradient. However, during pulmonary infection, auto- and/or paracrine-acting mediators induce pathophysiological changes of the alveolar–capillary barrier, altered expression of epithelial Na,K-ATPase and of epithelial ion channels including epithelial sodium channel and cystic fibrosis membrane conductance regulator, leading to the accumulation of edema and impaired alveolar fluid clearance. These mediators include classical pro-inflammatory cytokines such as TGF-β, TNF-α, interferons, or IL-1β that are released upon bacterial challenge with *Streptococcus pneumoniae, Klebsiella pneumoniae*, or *Mycoplasma pneumoniae* as well as in viral infection with influenza A virus, pathogenic coronaviruses, or respiratory syncytial virus. Moreover, the pro-apoptotic mediator TNF-related apoptosis-inducing ligand, extracellular nucleotides, or reactive oxygen species impair epithelial ion channel expression and function. Interestingly, during bacterial infection, alterations of ion transport function may serve as an additional feedback loop on the respiratory inflammatory profile, further aggravating disease progression. These changes lead to edema formation and impair edema clearance which results in suboptimal gas exchange causing hypoxemia and hypercapnia. Recent preclinical studies suggest that modulation of the alveolar–capillary fluid homeostasis could represent novel therapeutic approaches to improve outcomes in infection-induced lung injury.

## Introduction

The major task of the respiratory tract is the exchange between inhaled atmospheric oxygen and carbon dioxide carried by the bloodstream, which is ensured by a thin but large surface area formed by type I and type II alveolar epithelial cells. Both the upper and the lower respiratory epithelia are lined by a thin (0.2 µM) aqueous layer (1), referred to as airway surface liquid (ASL) and alveolar lining fluid (AFL), respectively. This fluidic component serves—in concerted action with surfactant, mucus, and ciliary beat—to reduce alveolar surface tension and prevent atelectasis as well as to defend against invading pathogens. To maintain the composition of the ASL and AFL and to prevent alveolar flooding, lung fluid homeostasis is tightly controlled by the expression and activity of ion channels and pumps. These channels and pumps establish an osmotic gradient between airspace and interstitium, driving paracellular or aquaporin- (AQP3, 4, and 5) (2) mediated fluid movement across the respiratory epithelium. Among these, the apical amiloride-sensitive epithelial sodium channel (ENaC) and the amiloride-insensitive cyclic nucleotide-gated cation channel (CNG) acting together with the basolaterally located Na,K-ATPase (NKA) promote transcellular sodium transport (3), which is accompanied in the alveolar epithelium by chloride uptake from the apical cystic fibrosis membrane conductance regulator (CFTR) (4). However, in the airway, CFTR promotes chloride secretion to regulate mucus density (5). In addition, Ca^2+^-activated ion channels (CaCC) promote apical chloride secretion, further supported by basolateral chloride uptake *via* Na^+^/K^+^/2Cl^−^ cotransporters (NKCC) (6) as well as potassium ion channels such as Kv7.1, contributing to cellular membrane potential and buildup of an electrochemical gradient necessary for apical chloride secretion (7). Additional factors influencing fluid homeostasis are epithelial (im)permeability established by tight junction proteins as well as endothelial integrity limiting the extravasation of fluid from the blood vessels driven by changes in the capillary hydrostatic pressure (8, 9).

Pulmonary infections commonly disturb ion and thus fluid homeostasis, resulting in abnormal changes of ASL, AFL, and alveolar edema formation. Both viral and bacterial pathogens are common causative agents for acute lung injury (ALI) and the acute respiratory distress syndrome (ARDS), which are characterized by a widespread inflammation within the lungs, extensive flooding of the alveolar airspace with protein-rich exudate fluid and impaired gas exchange leading to respiratory failure and resulting in mortality rates of 40–58% (10, 11). Additionally, sepsis resulting from primary infections at other sites is often complicated by the development of severe lung injury during the onset of bacteremia, resulting in lung failure and accounting for as many as half of all cases of ARDS (12). Although some of the pathogen-derived effects on ion transport during lung injury have been reported to be caused directly by the pathogen–host cell interaction (13), accumulating evidence suggests that auto- and paracrine mediators of local and/or systemic inflammatory responses mounted upon pathogen recognition and replication induce—among other pathophysiological changes—impaired ion transport and alveolar fluid clearance (AFC), resulting in edema formation and persistence. Importantly, mortality in ARDS patients has repeatedly been found to correlate with persistence of alveolar edema (11, 14).

In this review, we will highlight advances in the understanding of how inflammatory responses in pulmonary infection affect ion transport, including common patterns and unique pathways activated by different respiratory pathogens, and how these mechanisms might be modulated to improve the outcomes of ARDS patients.

## Mediators Modulating Ion and Fluid Homeostasis

**Figure 1:**
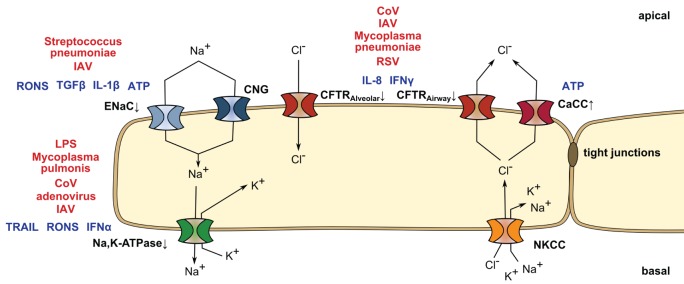
**Mediators released in pulmonary infection and their effects on ion homeostasis**. Ion transport of the lung epithelial cell is mediated by various ion channels and pumps. Sodium enters the epithelial cell *via* the apical cyclic nucleotide-gated cation channel (CNG) or the epithelial sodium channel (ENaC), that can be downregulated by reactive oxygen and nitrogen species (RONS) and ATP, transforming growth factor beta (TGF-β) or interleukin-1 beta (IL-1β) upon *Streptococcus pneumoniae* and influenza A virus (IAV) infection. Sodium is secreted at the basolateral side by the Na,K-ATPase (NKA), which is modulated in lipopolysaccharide (LPS)-induced lung injury as well as upon *Mycoplasma pulmonis*, IAV, coronavirus (CoV), or adenovirus challenge. RONS, interferon-alpha (IFN-α), and TNF-related apoptosis-inducing ligand (TRAIL) lead to a decrease in NKA abundance or activity. In parallel, chloride is taken up (alveolar epithelium) or secreted (airway) by the cystic fibrosis membrane conductance regulator (CFTR) and secreted by apical Ca^2+^-activated ion channels (CaCC), supported by basolateral potassium channels (not shown) and Na^+^/K^+^/2Cl^−^ cotransporters (NKCC). While extracellular ATP enhances chloride secretion by CaCC, CFTR action is reduced by IFN-γ and interleukin-8 (IL-8) in CoV, IAV, respiratory syncytial virus (RSV), or *Mycoplasma pneumoniae* infection.

There are numerous reports showing that pulmonary infection leads to loss of barrier integrity and edema accumulation as well as the role of distinct mediators on impairing ion channel or transporter function on the alveolar, bronchial, and gut epithelia. However, there have been few studies showing how infectious agents modulate soluble signaling molecules that affect ion and fluid homeostasis. Several reports from the last decade have reestablished an important role for soluble, inflammatory mediators in the progression of ARDS. For example, Lee et al. demonstrated that exposure of human ATII cells to pulmonary edema fluid derived from ARDS patients alone was sufficient to downregulate the ion channels and pumps involved in AFC, including ENaC, the NKA, and CFTR (15). Concomitantly, it was established that viral or bacterial lung infections lead to edema accumulation and impair clearance *via* the induction of paracrine factors. For example, influenza A virus (IAV) has been shown to increase apical potassium secretion by upregulation of the apical potassium channel KCNN4 by a paracrine signaling event, thus disturbing the osmotic gradient necessary for edema clearance (16). Similarly, *Pseudomonas aeruginosa* evokes a strong inflammatory response and lung edema accumulation related with the modulation of ENaC subunit expression (17, 18). In the next paragraphs, we will provide an overview on interconnections of mediators released in pulmonary infection and their effects on ion and fluid homeostasis (Figure 1).

### Interferon

Once cells detect pathogens by their specific and specialized pattern recognition receptors, they produce interferons (IFN), which can be detected—if not actively suppressed by a given pathogen—in most pulmonary infection scenarios. Effects of IFN on fluid homeostasis seem to be mostly limited to gamma IFN (IFN-γ), which have been attributed a modulatory role in both innate and adaptive immunity (19, 20). IFN-γ has been reported to decrease sodium transport at levels as low as 10 U/ml (21). Moreover, IFN-γ can also directly decrease chloride currents along the bronchial epithelium by downregulating CFTR due to a posttranscriptional modulation of CFTR messenger RNA (mRNA) stability and thus half-life (21–23). In contrast, both class I IFN, IFN-α, and IFN-β that are usually implicated in mounting a direct cellular pathogen-restrictive response do not modulate CFTR mRNA or protein abundance (22). IFN-α appears to negatively impact NKA cell membrane protein abundance during IAV infection *via* activating the metabolic sensor AMP-kinase (AMPK) (24). However, to date, there is no data supporting whether this effect of IFN-α on ion transport is a generalized response during pulmonary infections.

### Tumor Necrosis Factor Alpha (TNF-α)

Tumor necrosis factor alpha is a classical cytokine produced upon local or systemic inflammation, regulating differential processes such as proliferation and differentiation of immune cells as well as cell death (25–27). After initial conflicting studies, it has by now become clear that it plays a dichotomic role in lung fluid reabsorption (28). On one hand, TNF-α ligation to its receptor TNF receptor 1 (TNFR1, also named CD120a or p55) inhibits ENaC activity both *in vitro* and *in vivo via* a PKC-dependent mechanism (29). On the other hand, a distinct lectin-like domain of TNF different from the receptor-binding domain, which can be mimicked by the 17-amino acid circular TIP peptide (30), has been reported to increase edema reabsorption in rat bacterial pneumonia (31). Application of the TIP peptide has been demonstrated to elevate ENaC expression and open probability (32) resulting in enhanced AFC in *P. aeruginosa*-treated rats *in vivo* (31) and has furthermore been reported to increase NKA activity (33). In addition to its direct effects on ion channels and pumps of the alveolar epithelium, the TNF-α/TNFR1 interaction also modulates the integrity of the alveolar barrier, as it increases endothelial expression of chemoattractants and adhesion molecules including the interleukin-8 (IL-8; formerly called neutrophil chemotactic factor)/IL-8-receptor 2 axis, the intercellular adhesion molecule-1, platelet endothelial cell adhesion molecule-1, and vascular adhesion molecule-1, and thus promotes excessive recruitment of mononuclear phagocytes and neutrophils during lung inflammation (30, 34, 35). Importantly, besides cellular transmigration itself, neutrophil-derived proteases and neutrophil extracellular traps are central drivers of both endothelial and epithelial injury (36).

### Interleukin-1 Beta (IL-1β)

Interleukin-1 beta is one of the most commonly found cytokines in pulmonary edema and bronchoalveolar lavage fluids in experimental and human ARDS (37, 38) and is, for example, induced during *Klebsiella pneumoniae* bacterial pneumonia (39–41). It is mainly produced by macrophages and, similarly to TNF-α, has a major impact on cell proliferation, differentiation, and cell death. In pulmonary inflammation, IL-1β increases lung barrier permeability in *in vitro* and *in vivo* models of ARDS (41, 42) and may contribute to alveolar edema in lung injury models by impairing fluid reabsorption from the lungs. This can in part be attributed to decreased sodium absorption due to a decrease in αENaC expression and trafficking to the apical membrane of ATII cells (43). In addition, IL-1β in *Streptococcus pneumonia* infection (44)—and also TNF-α and IFN-γ (45)—can influence ion transport processes *via* activation of the pro-coagulant factors (46). Thrombin in particular has been demonstrated to impair AFC by increasing the PKC-ζ-dependent endocytosis of the alveolar NKA (47).

### Interleukin-8

Interleukin-8 is a chemotactic factor that correlates with neutrophil accumulation in distal airspaces of patients with ARDS and is a predictor of mortality (48–50). IL-8 is secreted by bronchial epithelial cells and can be induced by *Mycoplasma pneumoniae* antigen or live *M. pneumoniae* (51) as well as by severe acute respiratory syndrome coronavirus spike protein or respiratory syncytial virus infection (52, 53). The rate of AFC is impaired by high levels of IL-8 and is significantly lower in patients who have a pulmonary edema fluid concentration of IL-8 above 4,000 pg/ml (54). Mechanistically, IL-8 inhibits beta-2 adrenergic receptor (β2AR) agonist-stimulated fluid transport across rat and human alveolar epithelia. This inhibition is mediated by a PI3K-dependent desensitization and downregulation of the β2AR from the cell membrane associated with an inhibition of cyclic AMP generation normally observed in response to β2AR agonist stimulation (54).

### Transforming Growth Factor Beta (TGF-β)

The cytokine TGF-β is a critical factor for the development of ARDS. Besides its established role in dampening inflammatory responses (55), e.g., by driving macrophages toward an anti-inflammatory phenotype (56), it increases alveolar epithelial permeability to promote edema formation upon lipopolysaccharide (LPS) stimulation (57). Furthermore, TGF-β has been shown to inhibit amiloride-sensitive sodium transport by an ERK1/2-dependent inhibition of the αENaC subunit promoter activity, decreasing αENaC mRNA and protein expression (58). In addition, Peters et al. (59) demonstrated that TGF-β leads to the subsequent activation of phospholipase D1, phosphatidyl-inositol-4-phosphate 5-kinase 1α, and NADPH oxidase 4 (Nox4). Nox4 activation results in the production of reactive oxygen species (ROS) that in turn reduce cell surface stability of the αβγENaC complex and thus promote edema fluid accumulation. Moreover, TGF-β decreases NKA β1 subunit expression, resulting in decreased NKA activity in lung epithelial cells (60, 61). In further support of a role for TGF-β in lung injury, TGF-β levels are increased in lung fluids from patients with ALI/ARDS (62) and in murine models of *Streptococcus pneumoniae* and IAV infection (63, 64). Of note, TGF-β has been proposed to further aggravate edema formation in IAV infection by increasing epithelial cell death, causing a disruption of epithelial barrier integrity (64). Moreover, it has been implicated in the upregulation of cellular adhesins which increase host susceptibility to bacterial co-infections (65) posing a major risk for increased viral pneumonia-associated morbidity and mortality during influenza epidemics (66).

### TNF-Related Apoptosis-Inducing Ligand (TRAIL)

The principal role of TRAIL, highly released by lung macrophages upon viral infection, is to drive infected cells into apoptosis to limit pathogen spread. TRAIL has been reported to be produced especially during viral respiratory infections, including IAV-, adenovirus-, and paramyxovirus infection, and cell sensitivity to TRAIL-induced apoptosis is enhanced in infected cells by increased TRAIL-receptor expression (67, 68). However, this process also affects alveolar epithelial barrier integrity leading to edema accumulation (67, 69). Moreover, TRAIL signaling leads to NKA downregulation in IAV infection in non-infected neighboring alveolar epithelial cells mediated by AMPK (24). Accordingly, TRAIL signaling reduces AFC and promotes edema formation. In addition, TRAIL release upon IAV infection further favors bacterial superinfection with *S. pneumoniae*, aggravating lung injury (70).

### Nucleotides

During acute infection, extracellular nucleotides often serve as danger signals involved in recognition and control of pathogens by promoting the recruitment of inflammatory cells, stimulating pro-inflammatory cytokines, and increasing the production of ROS or nitric oxide (NO) (71, 72). Extracellular ATP, which can be released from the airway epithelia and is produced by endothelial cells upon acute inflammation, binds to P2 purinergic receptors to promote a calcium signaling-dependent stimulation of CaCC and a decreased open probability of ENaC (73, 74). Moreover, extracellular adenosine, produced from ATP by hydrolysis *via* the ecto-5′-nucleotidase CD73, is increased in bronchoalveolar lavage fluid of IAV-infected mice, and genetic deletion of the A1-adenosine-receptor is protective (75). However, CD73 is only to a limited extent involved in the progression of lung injury and has no effect on pulmonary edema formation (76).

### Reactive Oxygen and Nitrogen Species (RONS)

Reactive oxygen and nitrogen species have been shown to be involved in the development of epithelial injury in pathologic situations, including LPS-/sepsis-induced lung injury as well as viral pneumonia, in which RONS are produced in large quantities by alveolar phagocytes (77). Studies in rabbit and piglet lungs further elucidated that RONS affect AFC and edema persistence by inhibiting both the activity of ENaC and alveolar epithelial NKA (78, 79).

## Effects of Ion Changes on Cytokine Production

To add to the complexity of airway and alveolar fluid regulation, it has been suggested that not only ion channels, pumps, and transporters are modulated by signaling factors released upon pulmonary infection but also changes in ion transport influence the respiratory inflammatory response. For example, the transporter NKCC1—which plays a critical role in basolateral ion transport—can affect the severity of pneumonia and sepsis and consequently severity of lung injury, by regulating the ability of the alveolar–capillary barrier to modulate neutrophil infiltration into the air spaces of the lung (80). Lack of NKCC1 in a mouse model of pneumonia infection with *K. pneumonia* or LPS resulted in increased numbers of neutrophils in the lavage fluid, decreased bacteremia, and importantly mortality. It has, therefore, been suggested that the activity of NKCC1 contributes to edema formation and decreased neutrophil migration into the lung air spaces, probably contributing to reduce bacterial killing and the subsequent development of severe sepsis (81–83). Similarly, mutations of CFTR can amplify lung inflammation by upregulating pro-inflammatory responses caused by an increase in cytokine production upon NFκB activation in lung epithelial cells (84). Lack of functional neutrophilic CFTR in a model of LPS-induced lung inflammation contributes to inflammatory imbalance with NFκB translocation and a reduction of anti-inflammatory cytokines such as IL-10, favoring the increase in lung vascular permeability (85). Also ion imbalances in response to expression of viral ion channels or viroporins, has been recognized as potential pathogen recognition pathway that favors inflammasome activation and the release of IL-1β, TNF, and IL-6, which might contribute to the limitation of virus spreading (86, 87).

## Therapeutic Modulation of the Alveolar–Capillary Fluid Balance During Pulmonary Infection

As stated above, pulmonary infections—especially in severe cases—can lead to lung edema accumulation and impaired edema clearance. Lung edema results in impaired oxygenation and organ dysfunction which if not resolved leads to high mortality of patients with ARDS (11, 14). Current treatment options for infection-induced ARDS include antivirals and antibiotics. However, there is increased antibiotic resistance—reported for pathogens such as *K. pneumoniae, Escherichia coli, Staphylococcus aureus* and *P. aeruginosa* (82, 83, 88)—or lack of readily available treatment options for some acute emerging agents such as zoonotic influenza viruses or middle east respiratory syndrome coronavirus (89–91). Current approaches to treat ARDS patients include low tidal volume mechanical ventilation, positive end expiratory pressure, fluid management, and extracorporeal membrane oxygenation as measures to primarily improve oxygenation (92). Interestingly, lung-protective ventilation strategies have not only been reported to reduce mortality by 22% in patients with ARDS but also to diminish the number of neutrophils and the concentration of pro-inflammatory cytokines released in patient lavage fluids.

Novel approaches targeting host mediators known to promote lung edema formation and impair clearance such as studies on TIP peptide [see Tumor Necrosis Factor Alpha (TNF-α) above] administration in ARDS are being studied. Initial reports showed that AP301, a synthetic peptide mimicking TIP, induces ENaC activity in type II alveolar epithelial cells from dogs, pigs, and rats (93) and improves lung function in a porcine lung injury model (94). A subsequent phase II clinical trial with AP301 in ventilated ARDS patients resulted in improved AFC and oxygenation of these patients (95). Also, mesenchymal stem cells, which have been reported to improve epithelial barrier integrity in human AEC II treated with a cytokine mix composed of a combination of IL-1β, TNFα, and IFNγ (96), are currently tested for safety and efficacy in phase II trials (clinical trial identifiers NCT02097641, NCT01775774, NCT02112500). Studies on β2-agonists, which had been previously shown to improve vectorial sodium transport and edema clearance (97, 98), did not improve ARDS outcomes (99, 100), possibly due to an enhanced inflammatory response driven by lung macrophages (101). Further treatment options targeting para- or autocrine signaling events affecting AFC in preclinical models include glucocorticoids that suppress inflammation and upregulate both NKA (102) and ENaC (103, 104), neutralizing antibodies directed against virus-specific release of macrophage TRAIL that improve NKA expression as well as AFC in IAV-infected mice (24) and nitric oxide synthase inhibitors aminoguanidine or N(omega)-monomethyl-l-arginine (l-NMMA) that protect against pulmonary edema in LPS-induced lung injury as well as in IAV infection (77, 105).

## Conclusion

Pathogen-induced lung injury but also sepsis can lead to widespread respiratory inflammation that favors accumulation of lung edema leading to multiorgan dysfunction and poor outcomes. Recent advances in the development of novel treatment strategies targeting respiratory ion homeostasis show encouraging results, identifying them as promising candidates to improve AFC in ALI which could potentially improve the survival of patients with ARDS.

## Author Contributions

CP, SH, JS, and EL have performed bibliographic research and drafted the manuscript.

## Conflict of Interest Statement

The authors declare that the research was conducted in the absence of any commercial or financial relationships that could be construed as a potential conflict of interest.

## Abbreviations

AFC, alveolar fluid clearance; ALF, alveolar lining fluid; ALI, acute lung injury; AMPK, AMP-kinase; AQP, aquaporin; ARDS, acute respiratory distress syndrome; ASL, airway surface liquid; CaCC, Ca^2+^-activated ion channels; cAMP, cyclic AMP; CFTR, cystic fibrosis membrane conductance regulator; CNG, cyclic nucleotide-gated cation channel; ENaC, epithelial sodium channel; IAV, influenza A virus; ICAM, intercellular adhesion molecule-1; IFN, interferons; IL, interleukin; l-NMMA, N(omega)-monomethyl-l-arginine; LPS, lipopolysaccharide; MERS-CoV, middle east respiratory syndrome coronavirus; mRNA, messenger RNA; NETs, neutrophil extracellular traps; NKCC, Na^+^/K^+^/2Cl^−^ cotransporters; NO, nitric oxide; PECAM, platelet endothelial cell adhesion molecule-1; RONS, reactive oxygen and nitrogen species; ROS, reactive oxygen species; RSV, respiratory syncytial virus; SARS-CoV, severe acute respiratory syndrome coronavirus; TGF-β, transforming growth factor beta; TNFR1, TNF receptor 1; TNF-α, tumor necrosis factor alpha; TRAIL, TNF-related apoptosis-inducing ligand; VCAM, vascular adhesion molecule-1; β2AR, beta-2 adrenergic receptor.

## References

[1] Bastacky J Lee CY Goerke J Koushafar H Yager D Kenaga L Alveolar lining layer is thin and continuous: low-temperature scanning electron microscopy of rat lung. J Appl Physiol (1995) 79:1615–28. 8594022 10.1152/jappl.1995.79.5.1615

[2] Verkman AS Matthay MA Song Y. Aquaporin water channels and lung physiology. Am J Physiol Lung Cell Mol Physiol (2000) 278:L867–79. 10781416 10.1152/ajplung.2000.278.5.L867

[3] Bertorello AM Komarova Y Smith K Leibiger IB Efendiev R Pedemonte CH Analysis of Na+,K+-ATPase motion and incorporation into the plasma membrane in response to G protein-coupled receptor signals in living cells. Mol Biol Cell (2003) 14:1149–57. 10.1091/mbc.E02-06-0367 12631730 PMC151586

[4] Mutlu GM Adir Y Jameel M Akhmedov AT Welch L Dumasius V Interdependency of beta-adrenergic receptors and CFTR in regulation of alveolar active Na+ transport. Circ Res (2005) 96:999–1005. 10.1161/01.RES.0000164554.21993.AC 15802612

[5] Schwiebert EM Kizer N Gruenert DC Stanton BA. GTP-binding proteins inhibit cAMP activation of chloride channels in cystic fibrosis airway epithelial cells. Proc Natl Acad Sci U S A (1992) 89:10623–7. 10.1073/pnas.89.22.10623 1279687 PMC50393

[6] Fischer H Illek B Finkbeiner WE Widdicombe JH. Basolateral Cl channels in primary airway epithelial cultures. Am J Physiol Lung Cell Mol Physiol (2007) 292:L1432–43. 10.1152/ajplung.00032.2007 17322286

[7] Mall M Gonska T Thomas J Schreiber R Seydewitz HH Kuehr J Modulation of Ca^2+^-activated Cl- secretion by basolateral K+ channels in human normal and cystic fibrosis airway epithelia. Pediatr Res (2003) 53:608–18. 10.1203/01.PDR.0000057204.51420.DC 12612194

[8] Koval M. Claudin heterogeneity and control of lung tight junctions. Annu Rev Physiol (2013) 75:551–67. 10.1146/annurev-physiol-030212-183809 23072447

[9] Guidot DM Folkesson HG Jain L Sznajder JI Pittet JF Matthay MA. Integrating acute lung injury and regulation of alveolar fluid clearance. Am J Physiol Lung Cell Mol Physiol (2006) 291:L301–6. 10.1152/ajplung.00153.2006 16698856

[10] Brun-Buisson C Minelli C Bertolini G Brazzi L Pimentel J Lewandowski K Epidemiology and outcome of acute lung injury in European intensive care units. Results from the ALIVE study. Intensive Care Med (2004) 30:51–61. 10.1007/s00134-003-2136-x 14569423

[11] Matthay MA Zemans RL. The acute respiratory distress syndrome: pathogenesis and treatment. Annu Rev Pathol (2011) 6:147–63. 10.1146/annurev-pathol-011110-130158 20936936 PMC3108259

[12] Matthay MA Ware LB Zimmerman GA. The acute respiratory distress syndrome. J Clin Invest (2012) 122:2731–40. 10.1172/JCI60331 22850883 PMC3408735

[13] Londino JD Lazrak A Noah JW Aggarwal S Bali V Woodworth BA Influenza virus M2 targets cystic fibrosis transmembrane conductance regulator for lysosomal degradation during viral infection. FASEB J (2015) 29:2712–25. 10.1096/fj.14-268755 25795456 PMC4478808

[14] Sznajder JI Alveolar edema must be cleared for the acute respiratory distress syndrome patient to survive. Am J Respir Crit Care Med (2001) 163:1293–4. 10.1164/ajrccm.163.6.ed1801d 11371384

[15] Lee JW Fang X Dolganov G Fremont RD Bastarache JA Ware LB Acute lung injury edema fluid decreases net fluid transport across human alveolar epithelial type II cells. J Biol Chem (2007) 282:24109–19. 10.1074/jbc.M700821200 17580309 PMC2765119

[16] Waugh T Ching JC Zhou Y Loewen ME. Influenza A virus (H1N1) increases airway epithelial cell secretion by up-regulation of potassium channel KCNN4. Biochem Biophys Res Commun (2013) 438:581–7. 10.1016/j.bbrc.2013.08.012 23954634

[17] Morissette C Skamene E Gervais F Endobronchial inflammation following *Pseudomonas aeruginosa* infection in resistant and susceptible strains of mice. Infect Immun (1995) 63:1718–24. 7729877 10.1128/iai.63.5.1718-1724.1995 PMC173215

[18] Dagenais A Gosselin D Guilbault C Radzioch D Berthiaume Y. Modulation of epithelial sodium channel (ENaC) expression in mouse lung infected with *Pseudomonas aeruginosa*. Respir Res (2005) 6:2. 10.1186/1465-9921-6-2 15636635 PMC546414

[19] Schoenborn JR Wilson CB. Regulation of interferon-gamma during innate and adaptive immune responses. Adv Immunol (2007) 96:41–101. 10.1016/S0065-2776(07)96002-2 17981204

[20] Young HA Hardy KJ. Role of interferon-gamma in immune cell regulation. J Leukoc Biol (1995) 58:373–81. 7561512

[21] Galietta LJ Folli C Marchetti C Romano L Carpani D Conese M Modification of transepithelial ion transport in human cultured bronchial epithelial cells by interferon-gamma. Am J Physiol Lung Cell Mol Physiol (2000) 278:L1186–94. 10835324 10.1152/ajplung.2000.278.6.L1186

[22] Besancon F Przewlocki G Baro I Hongre AS Escande D Edelman A. Interferon-gamma downregulates CFTR gene expression in epithelial cells. Am J Physiol (1994) 267:C1398–404. 7526699 10.1152/ajpcell.1994.267.5.C1398

[23] Resta-Lenert S Barrett KE. Probiotics and commensals reverse TNF-alpha- and IFN-gamma-induced dysfunction in human intestinal epithelial cells. Gastroenterology (2006) 130:731–46. 10.1053/j.gastro.2005.12.015 16530515

[24] Peteranderl C Morales-Nebreda L Selvakumar B Lecuona E Vadász I Morty RE Macrophage-epithelial paracrine crosstalk inhibits lung edema clearance during influenza infection. J Clin Invest (2016) 126:1566–80. 10.1172/JCI83931 26999599 PMC4811131

[25] Gallipoli P Pellicano F Morrison H Laidlaw K Allan EK Bhatia R Autocrine TNF-alpha production supports CML stem and progenitor cell survival and enhances their proliferation. Blood (2013) 122:3335–9. 10.1182/blood-2013-02-485607 24041577 PMC3953090

[26] Gaur U Aggarwal BB. Regulation of proliferation, survival and apoptosis by members of the TNF superfamily. Biochem Pharmacol (2003) 66:1403–8. 10.1016/S0006-2952(03)00490-8 14555214

[27] Micheau O Tschopp J. Induction of TNF receptor I-mediated apoptosis via two sequential signaling complexes. Cell (2003) 114:181–90. 10.1016/S0092-8674(03)00521-X 12887920

[28] Braun C Hamacher J Morel DR Wendel A Lucas R. Dichotomal role of TNF in experimental pulmonary edema reabsorption. J Immunol (2005) 175:3402–8. 10.4049/jimmunol.175.5.3402 16116234

[29] Yamagata T Yamagata Y Nishimoto T Hirano T Nakanishi M Minakata Y The regulation of amiloride-sensitive epithelial sodium channels by tumor necrosis factor-alpha in injured lungs and alveolar type II cells. Respir Physiol Neurobiol (2009) 166:16–23. 10.1016/j.resp.2008.12.008 19150416

[30] Narasaraju T Yang E Samy RP Ng HH Poh WP Liew AA Excessive neutrophils and neutrophil extracellular traps contribute to acute lung injury of influenza pneumonitis. Am J Pathol (2011) 179:199–210. 10.1016/j.ajpath.2011.03.013 21703402 PMC3123873

[31] Rezaiguia S Garat C Delclaux C Meignan M Fleury J Legrand P Acute bacterial pneumonia in rats increases alveolar epithelial fluid clearance by a tumor necrosis factor-alpha-dependent mechanism. J Clin Invest (1997) 99:325–35. 10.1172/JCI119161 9006001 PMC507800

[32] Czikora I Alli A Bao HF Kaftan D Sridhar S Apell HJ A novel tumor necrosis factor-mediated mechanism of direct epithelial sodium channel activation. Am J Respir Crit Care Med (2014) 190:522–32. 10.1164/rccm.201405-0833OC 25029038 PMC4214088

[33] Vadász I Schermuly RT Ghofrani HA Rummel S Wehner S Mühldorfer I The lectin-like domain of tumor necrosis factor-[alpha] improves alveolar fluid balance in injured isolated rabbit lungs. Crit Care Med (2008) 36:1543–50. 10.1097/CCM.0b013e31816f485e 18434905

[34] Herold S von Wulffen W Steinmueller M Pleschka S Kuziel WA Mack M Alveolar epithelial cells direct monocyte transepithelial migration upon influenza virus infection: impact of chemokines and adhesion molecules. J Immunol (2006) 177:1817–24. 10.4049/jimmunol.177.3.1817 16849492

[35] Hammond ME Lapointe GR Feucht PH Hilt S Gallegos CA Gordon CA IL-8 induces neutrophil chemotaxis predominantly via type I IL-8 receptors. J Immunol (1995) 155:1428–33. 7636208

[36] Herold S Gabrielli NM Vadasz I. Novel concepts of acute lung injury and alveolar-capillary barrier dysfunction. Am J Physiol Lung Cell Mol Physiol (2013) 305:L665–81. 10.1152/ajplung.00232.2013 24039257

[37] Bauer TT Montón C Torres A Cabello H Fillela X Maldonado A Comparison of systemic cytokine levels in patients with acute respiratory distress syndrome, severe pneumonia, and controls. Thorax (2000) 55:46–52. 10.1136/thorax.55.1.46 10607801 PMC1745592

[38] Hoshino T Okamoto M Sakazaki Y Kato S Young HA Aizawa H. Role of proinflammatory cytokines IL-18 and IL-1beta in bleomycin-induced lung injury in humans and mice. Am J Respir Cell Mol Biol (2009) 41:661–70. 10.1165/rcmb.2008-0182OC 19265174 PMC10283344

[39] Olman MA White KE Ware LB Simmons WL Benveniste EN Zhu S Pulmonary edema fluid from patients with early lung injury stimulates fibroblast proliferation through IL-1β-induced IL-6 expression. J Immunol (2004) 172:2668–77. 10.4049/jimmunol.172.4.2668 14764742

[40] Sordi R Menezes-de-Lima O Della-Justina AM Rezende E Assreuy J. Pneumonia-induced sepsis in mice: temporal study of inflammatory and cardiovascular parameters. Int J Exp Pathol (2013) 94:144–55. 10.1111/iep.12016 23441627 PMC3607143

[41] Herold S Tabar TS Janssen H Hoegner K Cabanski M Lewe-Schlosser P Exudate macrophages attenuate lung injury by the release of IL-1 receptor antagonist in Gram-negative pneumonia. Am J Respir Crit Care Med (2011) 183:1380–90. 10.1164/rccm.201009-1431OC 21278303

[42] Lee YM Hybertson BM Cho HG Terada LS Cho O Repine AJ Platelet-activating factor contributes to acute lung leak in rats given interleukin-1 intratracheally. Am J Physiol Lung Cell Mol Physiol (2000) 279:L75–80. 10893205 10.1152/ajplung.2000.279.1.L75

[43] Roux J Kawakatsu H Gartland B Pespeni M Sheppard D Matthay MA Interleukin-1β decreases expression of the epithelial sodium channel α-subunit in alveolar epithelial cells via a p38 MAPK-dependent signaling pathway. J Biol Chem (2005) 280:18579–89. 10.1074/jbc.M410561200 15755725

[44] Yang H Ko HJ Yang JY Kim JJ Seo SU Park SG Interleukin-1 promotes coagulation, which is necessary for protective immunity in the lung against *Streptococcus pneumoniae* infection. J Infect Dis (2013) 207:50–60. 10.1093/infdis/jis651 23100560

[45] Bastarache JA Wang L Geiser T Wang Z Albertine KH Matthay MA The alveolar epithelium can initiate the extrinsic coagulation cascade through expression of tissue factor. Thorax (2007) 62:608–16. 10.1136/thx.2006.063305 17356058 PMC2117249

[46] Idell S. Coagulation, fibrinolysis, and fibrin deposition in acute lung injury. Crit Care Med (2003) 31:S213–20. 10.1097/01.CCM.0000057846.21303.AB 12682443

[47] Vadász I Morty RE Olschewski A Königshoff M Kohstall MG Ghofrani HA Thrombin impairs alveolar fluid clearance by promoting endocytosis of Na+,K+-ATPase. Am J Respir Cell Mol Biol (2005) 33:343–54. 10.1165/rcmb.2004-0407OC 16014898

[48] Kurdowska A Miller EJ Noble JM Baughman RP Matthay MA Brelsford WG Anti-IL-8 autoantibodies in alveolar fluid from patients with the adult respiratory distress syndrome. J Immunol (1996) 157:2699–706. 8805676

[49] Pease J Sabroe I The role of interleukin-8 and its receptor in inflammatory lung disease: implications for therapy. Am J Respir Med (2002) 1:19–25. 10.1007/BF03257159 14720072 PMC7102088

[50] Goodman RB Strieter RM Martin DP Steinberg KP Milberg JA Maunder RJ Inflammatory cytokines in patients with persistence of the acute respiratory distress syndrome. Am J Respir Crit Care Med (1996) 154:602–11. 10.1164/ajrccm.154.3.8810593 8810593

[51] Chen Z Shao X Dou X Zhang X Wang Y Zhu C Role of the *Mycoplasma pneumoniae*/interleukin-8/neutrophil axis in the pathogenesis of pneumonia. PLoS One (2016) 11:e0146377. 10.1371/journal.pone.0146377 26752656 PMC4708980

[52] Chang YJ Liu CY Chiang BL Chao YC Chen CC. Induction of IL-8 release in lung cells via activator protein-1 by recombinant baculovirus displaying severe acute respiratory syndrome-coronavirus spike proteins: identification of two functional regions. J Immunol (2004) 173:7602–14. 10.4049/jimmunol.173.12.7602 15585888

[53] Redondo E Gazquez A Vadillo S Garcia A Franco A Masot AJ. Induction of interleukin-8 and interleukin-12 in neonatal ovine lung following experimental inoculation of bovine respiratory syncytial virus. J Comp Pathol (2014) 150:434–48. 10.1016/j.jcpa.2013.08.002 24854063

[54] Roux J McNicholas CM Carles M Goolaerts A Houseman BT Dickinson DA IL-8 inhibits cAMP-stimulated alveolar epithelial fluid transport via a GRK2/PI3K-dependent mechanism. FASEB J (2013) 27:1095–106. 10.1096/fj.12-219295 23221335 PMC3574281

[55] Shull MM Ormsby I Kier AB Pawlowski S Diebold RJ Yin M Targeted disruption of the mouse transforming growth factor-beta 1 gene results in multifocal inflammatory disease. Nature (1992) 359:693–9. 10.1038/359693a0 1436033 PMC3889166

[56] Gong D Shi W Yi SJ Chen H Groffen J Heisterkamp N TGFbeta signaling plays a critical role in promoting alternative macrophage activation. BMC Immunol (2012) 13:31 10.1186/1471-2172-13-31 22703233 PMC3406960

[57] Pittet JF Griffiths MJ Geiser T Kaminski N Dalton SL Huang X TGF-beta is a critical mediator of acute lung injury. J Clin Invest (2001) 107:1537–44. 10.1172/JCI11963 11413161 PMC200192

[58] Frank J Roux J Kawakatsu H Su G Dagenais A Berthiaume Y Transforming growth factor-beta1 decreases expression of the epithelial sodium channel alphaENaC and alveolar epithelial vectorial sodium and fluid transport via an ERK1/2-dependent mechanism. J Biol Chem (2003) 278:43939–50. 10.1074/jbc.M304882200 12930837

[59] Peters DM Vadasz I Wujak L Wygrecka M Olschewski A Becker C TGF-beta directs trafficking of the epithelial sodium channel ENaC which has implications for ion and fluid transport in acute lung injury. Proc Natl Acad Sci U S A (2014) 111:E374–83. 10.1073/pnas.1306798111 24324142 PMC3903252

[60] Wujak LA Becker S Seeger W Morty RE TGF-β regulates Na,K-ATPase activity by changing the regulatory subunit stoichiometry of the Na,K-ATPase complex. FASEB J (2011) 25(Suppl 1039.9). Available from: http://www.fasebj.org/content/25/1_Supplement/1039.9

[61] Wujak ŁA Blume A Baloğlu E Wygrecka M Wygowski J Herold S FXYD1 negatively regulates Na(+)/K(+)-ATPase activity in lung alveolar epithelial cells. Respir Physiol Neurobiol (2016) 220:54–61. 10.1016/j.resp.2015.09.008 26410457

[62] Budinger GRS Chandel NS Donnelly HK Eisenbart J Oberoi M Jain M Active transforming growth factor-β1 activates the procollagen I promoter in patients with acute lung injury. Intensive Care Med (2005) 31:121–8. 10.1007/s00134-004-2503-2 15565360 PMC7095267

[63] Neill DR Fernandes VE Wisby L Haynes AR Ferreira DM Laher A T regulatory cells control susceptibility to invasive pneumococcal pneumonia in mice. PLoS Pathog (2012) 8:e1002660. 10.1371/journal.ppat.1002660 22563306 PMC3334885

[64] Schultz-Cherry S Hinshaw VS. Influenza virus neuraminidase activates latent transforming growth factor beta. J Virol (1996) 70:8624–9. 8970987 10.1128/jvi.70.12.8624-8629.1996 PMC190955

[65] Li N Ren A Wang X Fan X Zhao Y Gao GF Influenza viral neuraminidase primes bacterial coinfection through TGF-beta-mediated expression of host cell receptors. Proc Natl Acad Sci U S A (2015) 112:238–43. 10.1073/pnas.1414422112 25535343 PMC4291618

[66] Rynda-Apple A Robinson KM Alcorn JF. Influenza and bacterial superinfection: illuminating the immunologic mechanisms of disease. Infect Immun (2015) 83:3764–70. 10.1128/IAI.00298-15 26216421 PMC4567631

[67] Hogner K Wolff T Pleschka S Plog S Gruber AD Kalinke U Macrophage-expressed IFN-beta contributes to apoptotic alveolar epithelial cell injury in severe influenza virus pneumonia. PLoS Pathog (2013) 9:e1003188 10.1371/journal.ppat.1003188 23468627 PMC3585175

[68] Kirshner JR Karpova AY Kops M Howley PM. Identification of TRAIL as an interferon regulatory factor 3 transcriptional target. J Virol (2005) 79:9320–4. 10.1128/JVI.79.14.9320-9324.2005 15994827 PMC1168760

[69] Herold S Steinmueller M von Wulffen W Cakarova L Pinto R Pleschka S Lung epithelial apoptosis in influenza virus pneumonia: the role of macrophage-expressed TNF-related apoptosis-inducing ligand. J Exp Med (2008) 205:3065–77. 10.1084/jem.20080201 19064696 PMC2605231

[70] Ellis GT Davidson S Crotta S Branzk N Papayannopoulos V Wack A. TRAIL+ monocytes and monocyte-related cells cause lung damage and thereby increase susceptibility to influenza-*Streptococcus pneumoniae* coinfection. EMBO Rep (2015) 16:1203–18. 10.15252/embr.201540473 26265006 PMC4576987

[71] Coutinho-Silva R Ojcius DM. Role of extracellular nucleotides in the immune response against intracellular bacteria and protozoan parasites. Microbes Infect (2012) 14:1271–7. 10.1016/j.micinf.2012.05.009 22634346 PMC4110109

[72] Savio LE Coutinho-Silva R Purinergic signaling in infection and autoimmune disease. Biomed J (2016) 39:304–5. 10.1016/j.bj.2016.09.002 27884376 PMC6138793

[73] Blaug S Rymer J Jalickee S Miller SS. P2 purinoceptors regulate calcium-activated chloride and fluid transport in 31EG4 mammary epithelia. Am J Physiol Cell Physiol (2003) 284:C897–909. 10.1152/ajpcell.00238.2002 12456394

[74] Pochynyuk O Bugaj V Vandewalle A Stockand JD. Purinergic control of apical plasma membrane PI(4,5)P2 levels sets ENaC activity in principal cells. Am J Physiol Renal Physiol (2008) 294:F38–46. 10.1152/ajprenal.00403.2007 17913833

[75] Wolk KE Lazarowski ER Traylor ZP Yu EN Jewell NA Durbin RK Influenza A virus inhibits alveolar fluid clearance in BALB/c mice. Am J Respir Crit Care Med (2008) 178:969–76. 10.1164/rccm.200803-455OC 18689466 PMC2577730

[76] Aeffner F Woods PS Davis IC. Ecto-5’-nucleotidase CD73 modulates the innate immune response to influenza infection but is not required for development of influenza-induced acute lung injury. Am J Physiol Lung Cell Mol Physiol (2015) 309:L1313–22. 10.1152/ajplung.00130.2015 26432867 PMC4669338

[77] Akaike T Noguchi Y Ijiri S Setoguchi K Suga M Zheng YM Pathogenesis of influenza virus-induced pneumonia: involvement of both nitric oxide and oxygen radicals. Proc Natl Acad Sci U S A (1996) 93:2448–53. 10.1073/pnas.93.6.2448 8637894 PMC39817

[78] Nielsen VG Baird MS Chen LAN Matalon S. DETANONOate, a nitric oxide donor, decreases amiloride-sensitive alveolar fluid clearance in rabbits. Am J Respir Crit Care Med (2000) 161:1154–60. 10.1164/ajrccm.161.4.9907033 10764305

[79] Youssef JA Thibeault DW Rezaiekhaligh MH Mabry SM Norberg MI Truog WE. Influence of inhaled nitric oxide and hyperoxia on Na,K-ATPase expression and lung edema in newborn piglets. Neonatology (1999) 75:199–209. 10.1159/000014096 9925907

[80] Matthay MA Su X Pulmonary barriers to pneumonia and sepsis. Nat Med (2007) 13:780–1. 10.1038/nm0707-780 17618264

[81] Nguyen M Pace AJ Koller BH. Mice lacking NKCC1 are protected from development of bacteremia and hypothermic sepsis secondary to bacterial pneumonia. J Exp Med (2007) 204:1383–93. 10.1084/jem.20061205 17517966 PMC2118609

[82] Nathan C Cars O Antibiotic resistance – problems, progress, and prospects. N Engl J Med (2014) 371:1761–3. 10.1056/NEJMp1408040 25271470

[83] Pendleton JN Gorman SP Gilmore BF. Clinical relevance of the ESKAPE pathogens. Expert Rev Anti Infect Ther (2013) 11:297–308. 10.1586/eri.13.12 23458769

[84] Blackwell TS Stecenko AA Christman JW Dysregulated NF-κB activation in cystic fibrosis: evidence for a primary inflammatory disorder. Am J Physiol Lung Cell Mol Physiol (2001) 281:L69–70. 11404247 10.1152/ajplung.2001.281.1.L69

[85] Su X Looney MR Su H Lee JW Song Y Matthay MA. Role of CFTR expressed by neutrophils in modulating acute lung inflammation and injury in mice. Inflamm Res (2011) 60:619–32. 10.1007/s00011-011-0313-x 21301926 PMC3116128

[86] Nieto-Torres JL DeDiego ML Verdiá-Báguena C Jimenez-Guardeño JM Regla-Nava JA Fernandez-Delgado R Severe acute respiratory syndrome coronavirus envelope protein ion channel activity promotes virus fitness and pathogenesis. PLoS Pathog (2014) 10:e1004077. 10.1371/journal.ppat.1004077 24788150 PMC4006877

[87] Triantafilou K Triantafilou M. Ion flux in the lung: virus-induced inflammasome activation. Trends Microbiol (2014) 22:580–8. 10.1016/j.tim.2014.06.002 24986075 PMC7126464

[88] Mizgerd JP Lung infection – a public health priority. PLoS Med (2006) 3:e76 10.1371/journal.pmed.0030076 16401173 PMC1326257

[89] Graham RL Donaldson EF Baric RS. A decade after SARS: strategies for controlling emerging coronaviruses. Nat Rev Microbiol (2013) 11:836–48. 10.1038/nrmicro3143 24217413 PMC5147543

[90] Zhu H Webby R Lam TT Smith DK Peiris JS Guan Y. History of Swine influenza viruses in Asia. Curr Top Microbiol Immunol (2013) 370:57–68. 10.1007/82_2011_179 21948002

[91] Zumla A Memish ZA Maeurer M Bates M Mwaba P Al-Tawfiq JA Emerging novel and antimicrobial-resistant respiratory tract infections: new drug development and therapeutic options. Lancet Infect Dis (2014) 14:1136–49. 10.1016/S1473-3099(14)70828-X 25189352 PMC7106460

[92] Gonzales JN Lucas R Verin AD. The acute respiratory distress syndrome: mechanisms and perspective therapeutic approaches. Austin J Vasc Med (2015) 2:1009. 26973981 PMC4786180

[93] Tzotzos S Fischer B Fischer H Pietschmann H Lucas R Dupré G AP301, a synthetic peptide mimicking the lectin-like domain of TNF, enhances amiloride-sensitive Na(+) current in primary dog, pig and rat alveolar type II cells. Pulm Pharmacol Ther (2013) 26:356–63. 10.1016/j.pupt.2012.12.011 23313096 PMC3646188

[94] Hartmann EK Boehme S Duenges B Bentley A Klein KU Kwiecien R An inhaled tumor necrosis factor-alpha-derived TIP peptide improves the pulmonary function in experimental lung injury. Acta Anaesthesiol Scand (2013) 57:334–41. 10.1111/aas.12034 23216436

[95] Krenn K Croize A Klein KU Böhme S Markstaller K Ullrich R Oral inhalation of AP301 peptide activates pulmonary oedema clearance: initial results from a phase IIa clinical trial in mechanically ventilated ICU patients. Eur Respir J (2014) 44:1386.

[96] Fang X Neyrinck AP Matthay MA Lee JW. Allogeneic human mesenchymal stem cells restore epithelial protein permeability in cultured human alveolar type II cells by secretion of angiopoietin-1. J Biol Chem (2010) 285:26211–22. 10.1074/jbc.M110.119917 20554518 PMC2924032

[97] Mutlu GM Dumasius V Burhop J McShane PJ Meng FJ Welch L Upregulation of alveolar epithelial active Na+ transport is dependent on beta2-adrenergic receptor signaling. Circ Res (2004) 94:1091–100. 10.1161/01.RES.0000125623.56442.20 15016730

[98] Mutlu GM Factor P. Alveolar epithelial beta2-adrenergic receptors. Am J Respir Cell Mol Biol (2008) 38:127–34. 10.1165/rcmb.2007-0198TR 17709598 PMC2214676

[99] Gao Smith F Perkins GD Gates S Young D McAuley DF Tunnicliffe W Effect of intravenous beta-2 agonist treatment on clinical outcomes in acute respiratory distress syndrome (BALTI-2): a multicentre, randomised controlled trial. Lancet (2012) 379:229–35. 10.1016/S0140-6736(11)61623-1 22166903 PMC3266479

[100] National Heart, Lung, and Blood Institute Acute Respiratory Distress Syndrome (ARDS) Clinical Trials Network Matthay MA Brower RG Carson S Douglas IS Eisner M Randomized, placebo-controlled clinical trial of an aerosolized beta(2)-agonist for treatment of acute lung injury. Am J Respir Crit Care Med (2011) 184:561–8. 10.1164/rccm.201012-2090OC 21562125 PMC3175548

[101] Chiarella SE Soberanes S Urich D Morales-Nebreda L Nigdelioglu R Green D Beta(2)-Adrenergic agonists augment air pollution-induced IL-6 release and thrombosis. J Clin Invest (2014) 124:2935–46. 10.1172/JCI75157 24865431 PMC4071386

[102] Barquin N Ciccolella DE Ridge KM Sznajder JI. Dexamethasone upregulates the Na-K-ATPase in rat alveolar epithelial cells. Am J Physiol (1997) 273:L825–30. 9357858 10.1152/ajplung.1997.273.4.L825

[103] Itani OA Auerbach SD Husted RF Volk KA Ageloff S Knepper MA Glucocorticoid-stimulated lung epithelial Na(+) transport is associated with regulated ENaC and sgk1 expression. Am J Physiol Lung Cell Mol Physiol (2002) 282:L631–41. 10.1152/ajplung.00085.2001 11880287

[104] Nakamura K Stokes JB McCray PB Jr. Endogenous and exogenous glucocorticoid regulation of ENaC mRNA expression in developing kidney and lung. Am J Physiol Cell Physiol (2002) 283:C762–72. 10.1152/ajpcell.00029.2002 12176733

[105] Heremans H Dillen C Groenen M Matthys P Billiau A. Role of interferon-gamma and nitric oxide in pulmonary edema and death induced by lipopolysaccharide. Am J Respir Crit Care Med (2000) 161:110–7. 10.1164/ajrccm.161.1.9902089 10619806

